# Isolation and Characterization of Novel Cellulose Micro/Nanofibers from *Lygeum spartum* Through a Chemo-Mechanical Process

**DOI:** 10.3390/polym16213001

**Published:** 2024-10-25

**Authors:** Sabrina Ahmima, Nacira Naar, Patryk Jędrzejczak, Izabela Klapiszewska, Łukasz Klapiszewski, Teofil Jesionowski

**Affiliations:** 1 Laboratory of Macromolecular Synthesis and Thio-Organics Macromolecular, Faculty of Chemistry, University of Sciences and Technology Houari Boumediene USTHB, BP 32, El Alia, Bab Ezzouar, Alger 16111, Algeria; sabrina.ahmima@gmail.com; 2 Institute of Chemical Technology and Engineering, Faculty of Chemical Technology, Poznan University of Technology, Berdychowo 4, PL-60965 Poznan, Poland; patryk.jedrzejczak@put.poznan.pl (P.J.); teofil.jesionowski@put.poznan.pl (T.J.); 3 Institute of Building Engineering, Faculty of Civil and Transport Engineering, Poznan University of Technology, Piotrowo 3, PL-60965 Poznan, Poland; izabela.klapiszewska@put.poznan.pl

**Keywords:** cellulose micro/nanofibers, *Lygeum spartum*, ultrasonication, homogenization

## Abstract

Recent studies have focused on the development of bio-based products from sustainable resources using green extraction approaches, especially nanocellulose, an emerging nanoparticle with impressive properties and multiple applications. Despite the various sources of cellulose nanofibers, the search for alternative resources that replace wood, such as *Lygeum spartum*, a fast-growing Mediterranean plant, is crucial. It has not been previously investigated as a potential source of nanocellulose. This study investigates the extraction of novel cellulose micro/nanofibers from *Lygeum spartum* using a two-step method, including both alkali and mechanical treatment as post-treatment with ultrasound, as well as homogenization using water and dilute alkali solution as a solvent. To determine the structural properties of CNFs, a series of characterization techniques was applied. A significant correlation was observed between the Fourier transform infrared (FTIR) spectroscopy and X-ray diffraction (XRD) results. The FTIR results revealed the elimination of amorphous regions and an increase in the energy of the H-bonding modes, while the XRD results showed that the crystal structure of micro/nanofibers was preserved during the process. In addition, they indicated an increase in the crystallinity index obtained with both methods (deconvolution and Segal). Thermal analysis based on thermogravimetric analysis (TGA) and differential scanning calorimetry (DSC) confirmed improvement in the thermal properties of the isolated micro/nanofibers. The temperatures of maximum degradation were 335 °C and 347 °C. Morphological analysis using a scanning electron microscope (SEM) and atomic force microscope (AFM) showed the formation of fibers along the axis, with rough and porous surfaces. The findings indicate the potential of *Lygeum spartum* as a source for producing high-quality micro/nanofibers. A future direction of study is to use the cellulose micro/nanofibers as additives in recycled paper and to evaluate the mechanical properties of the paper sheets, as well as investigate their use in smart paper.

## 1. Introduction

With growing awareness of environmental pollution and global warming, the development of sustainable solutions has become a crucial goal, with the aim of replacing petroleum products with environmentally friendly, bio-based materials. This strategy has encouraged the maximum use of naturally derived materials, and as a consequence, the global production of bio-based materials has increased over the last decade. In this context, polysaccharides are promising sources due to their availability from different resources, renewability, biodegradability, biocompatibility, and ease of processing [1]. Recently, significant research initiatives have been undertaken to prepare nanoscale particles from polysaccharides and to promote their application in various fields [2,3,4]. Among several biopolymer nanoparticles, nanocellulose (NC) is a class of futuristic materials that can be isolated from plant cell walls. This anisotropic nanoparticle has impressive thermal, mechanical, biological, and optical properties [5], which encourage its use in the development of new functional materials. Nanocellulose is isolated from various biomass resources by enzymatic, chemical, and mechanical methods [6,7]. Based on the preparation method, composition, morphology and dimension, and also depending on the source of the cellulose, types of NC are classified as cellulose nanocrystals (CNCs) or cellulose nanofibers (CNFs).

Researchers have made extensive use of the conventional acid hydrolysis extraction method; however, this method has drawbacks, such as equipment corrosion and high water consumption, due to the use of concentrated sulfuric acid, in addition to the lower thermal stability and difficulty in introducing new functional groups into NC due to the presence of sulfate groups [8]. On the other hand, CNFs are usually isolated by mechanical methods, including high-pressure homogenization, cryocrushing, and ultrasonication [9,10]. Besides the high energy consumption, the large mechanical forces can damage the CNFs, and the inability to control the size distribution of CNFs has limited the utilization of these extraction methods. For these reasons, there is a need to develop suitable, cost-effective nanocellulose extraction technologies. The combination of chemical methods with other mechanical and enzymatic techniques has been widely studied as a way to optimize the production of high-quality NC with enhanced properties [11,12]. Table 1 summarizes the methods of nanocellulose extraction from various sources.

Recently, ultrasonication has become one of the main emerging technologies for the conversion of lignocellulosic biomass into value-added products [13]. Extensive research has shown that ultrasonication preserves the biodegradability and biocompatibility of cellulose [14]. Ultrasound generates intense mechanical forces, such as shear forces and shock waves, creating acoustic cavitation. This results in a collapse, which generates 10–100 kJ mol^−1^ of energy, cleaving the hydrogen bond network that holds the fibrils together [15]. The coupling of ultrasound with other mechanical and chemical methods has shown advantages in NC production, where varieties of NC with different crystallinity indices, BET surface areas, and dimensions have been isolated by controlling sonication parameters such as time, solvent, temperature, and frequency. The ultrasonic approach has been combined with various chemical treatments to increase technical feasibility. Shi et al. [16] proposed isolating cellulose nanocrystals with alkali hydrolysis. Using sonication as a post-pretreatment, they found that the resulting CNC has a higher crystallinity index and thermal stability. Thus, the significant progress in nanotechnology has broadened the scope of research to include the development of new extraction approaches and the use of new resources, such as non-wood products. These are available in abundance due to their fast-growing cycle and offer a variety of properties, depending on their chemical composition; non-wood products also contain less lignin compared to wood.

In this context, the present work investigates, for the first time, the extraction of cellulose micro/nanofibers from *Lygeum spartum*, a fast-growing, typically Mediterranean grass, as a novel and sustainable source replacing the Alfa plant, a grass from the same family as *Lygeum spartum*, growing in the same region and extensively studied. A two-step energy-efficient extraction method (ultrasound and homogenization) was used to nano-size the cellulose [17], and alkali pretreatment was used to remove lignin and hemicellulose. In all nanocellulose production routes, water acts as a medium, and water molecules swell the fibers, opening their structure, increasing their accessibility, and facilitating the penetration of chemical reagents. For these reasons, a dilute sodium hydroxide solution was used during the homogenization and sonication steps. The chemical structure, microstructure, and morphology of the resulting micro/nanofibers were extensively evaluated using FTIR, XRD, SEM, and AFM, respectively. In particular, FTIR deconvolution in the 3800 to 3000 cm^−1^ region was used to study the evolution of inter- and intramolecular hydrogen-bonding energies. TGA and DSC analyses were performed to measure the thermal properties of the isolated micro/nanofibers. The present approach and the use of the Spart plant represent a promising avenue for nanocellulose extraction and applications in the field of nanocomposites.

**Table 1:** Preparation methods and applications of CNFs.

Classification	Preparation Methods	Application	References
CNF, CNC	acidic hydrolysis and steam explosion	nanocomposite fabrication	[18]
CNF	ultrasonication combined with high-pressure homogenization	industrial PCNFs	[19]
CNF	TEMPO-mediated oxidation, steam explosion	-	[20]
CNF	ultrasound-assisted method	hydrogel, gas sensors, and packaging materials	[21]

## 2. Materials and Methods

### 2.1. Materials

*Lygeum spartum* fibers collected in Algiers (Algeria) were used in this study. The chemical reagents ethanol (≥99.8%) and sodium hypochlorite were purchased from Sigma-Aldrich (St. Louis, MO, USA), and sodium hydroxide was supplied by Chempur (Piekary Śląskie, Poland).

### 2.2. Isolation of Cellulose Fiber from Lygeum spartum

*Lygeum spartum* fibers were washed with distilled water and air-dried, then ground into powder. These steps remove impurities from the fiber surface and improve extraction efficiency. To remove waxes and pectin, the powder was treated with a 2/1 (*v*/*v*) ethanol/water mixture overnight at room temperature with continuous stirring. This method replaces the conventional Soxhlet extraction process, which uses a mixture of toluene and ethanol. The dewaxed sample was washed several times with distilled water at 85 °C and oven-dried at 40 °C to eliminate lignin and hemicellulose. The dewaxed fibers were treated with 1 M NaOH solution for 2 h at 60 °C. The obtained cellulose was washed with distilled water until neutral pH was reached and bleached with sodium hypochlorite solution (5% for 10 g bleached cellulose) for 1 h at 60 °C to remove residual lignin. It was then washed several times with distilled water until neutral pH was reached, and was air-dried. The pristine cellulose was preserved in a zip bag to avoid moisture absorption.

### 2.3. Isolation of Cellulose Micro/Nanofibers from Lygeum spartum

**Figure 1:**
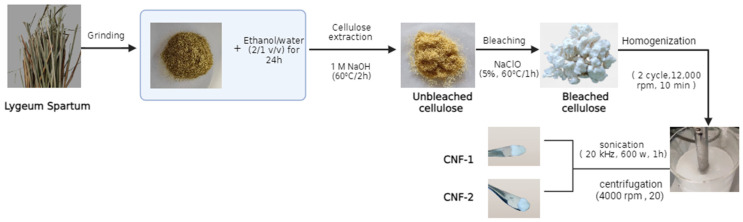
Steps in the preparation of cellulosic micro/nanofibers.

To evaluate the effect of the sonication parameter on CNF fibers, the cellulose fibers were diluted in distilled water (CNF-1) and in a diluted alkali solution (0.5 M) (CNF-2). The suspensions were treated twice with a homogenizer (IKA Ultra Turrax T25 digital, IKA-Werke GmbH & Co. KG, Staufen, Germany) at 2000 rpm for 10 min. The homogenized suspensions were processed by sonication at 20 kHz and 600 W for 1 h, followed by centrifugation at 4000 rpm for 20 min. The CNF-2 suspension was successively centrifuged with distilled water until a neutral pH was reached, to ensure the removal of free bases. The CNF fibers were dried for further analysis (see Figure 1).

### 2.4. Characterization of Materials

#### 2.4.1. Fourier Transform Infrared Spectroscopy (FTIR)

The chemical structure and functional groups present in the samples were analyzed with a Vertex 70 spectrometer from Bruker Optics GmbH & Co. KG (Ettlingen, Germany), and the FTIR spectra were recorded in the wavenumber range 4000–400 cm^−1^ at a resolution of 4 cm^−1^. To study the changes in hydrogen bonding in CNFs, the H-bonding characteristic parameters—energies (*E_H_*) and length (*R*)—for cellulose-LS, CNF-1, and CNF-2 in different H-bonding modes were calculated using Equations (1) and (2) [22], as follows:

(1) EH=1k×v0−vv0

where *v*_0_ is the standard frequency of free OH groups (3600 cm^−1^), *v* is the frequency of hydrogen-bonded hydroxyl groups, and *k* = 1.68·10^−2^/kcal. Deconvolution (in the region 3800–3000 cm^−1^) was performed using OriginLab Pro 2024b software with the Gaussian function

(2) $$\Delta_{v}=4.43\times 103\left(2.84-R\right)$$

where Δ*_v_* is the frequency shift in cm^−1^ (Δ*_v_* = *v*_0_ − *v*), and *R* is the band length (Å).

#### 2.4.2. X-Ray Diffraction (XRD)

To investigate the microstructure of the samples, XRD analysis was performed using a Smart Lab X-ray diffractometer (Rigaku, Tokyo, Japan) with Cu Kα radiation (λ = 1.54060 Å) in the angular range 5–50°, with a step size of 0.026°/2θ. The crystallinity index was calculated using Segal’s Equation (3) [23]:

(3) $$CrI=(\left(I_{200}-I_{am})/I_{200}\right)\times 100$$

where *I*_200_ is the maximum intensity of the crystalline regions (200), and *I_am_* is the minimum intensity of the amorphous regions.

The peak deconvolution method was used to evaluate the crystallinity of cellulose nanofibers [24]. Curve fitting was performed using the Gaussian fit in OriginLab Pro 2024b software. *CrI* was calculated using the following Equation (4):

(4) $$CrI=(A_{c}/\left(A_{c}+A_{a}\right))\times 100$$

where *A_c_* and *A_a_* are the areas under the crystalline and amorphous peaks, respectively.

The crystallite size of the samples was calculated for the crystallographic peak (200) using the Debye–Scherrer formula [25]. Furthermore, the d-spacing was calculated according to Bragg’s equation for the (200) plane.

#### 2.4.3. Thermal Stability

Thermal stability and degradation were analyzed using the Netzsch STA 449F3 Jupiter instrument (Netzsch, Selb, Germany). A sample of approximately 10 mg was heated at 10 °C/min over a temperature range from room temperature to 800 °C.

#### 2.4.4. Differential Scanning Calorimetry (DSC)

Differential scanning calorimetry was performed using a DSC Q200 coupled to a TA Instruments system (TA Instruments, Inc., New Castle, DE, USA) in an inert nitrogen atmosphere at a heating rate of 10 °C/min, over a temperature range from room temperature to 400 °C.

#### 2.4.5. Scanning Electron Microscopy (SEM)

Scanning electron microscope analysis was used to evaluate the microstructure of the samples. The images were taken with a Tescan VEGA3 instrument (Tescan Orsay Holding a.s., Brno, Czech Republic).

#### 2.4.6. Atomic Force Microscopy (AFM)

Images of the CNF surface morphology were obtained using a Bruker Dimension Icon (AFM, Bruker, Billerica, MA, USA). Atomic force microscopy (AFM) analysis was performed in tapping mode, with a scan rate of 1 Hz on CNF films.

## 3. Results and Discussion

### 3.1. Fourier Transform Infrared Spectroscopy

**Figure 2:**
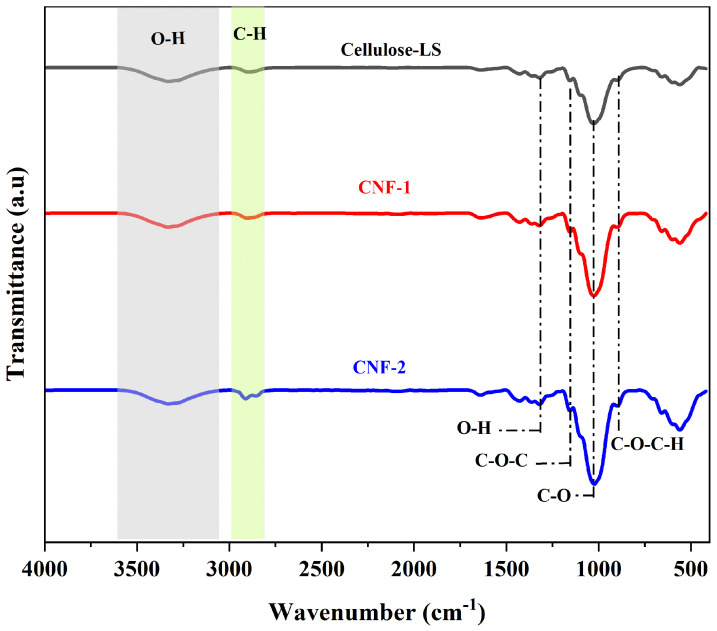
FTIR spectra of cellulose-LS, CNF-1, and CNF-2.

FTIR was used to study the functional group changes in the fibers after the treatments. The IR spectra of cellulose-LS, CNF-1, and CNF-2 are shown in Figure 2. Two main absorption regions were observed in the spectrum, within the ranges of 3600–2800 cm^−1^ and 1640–750 cm^−1^. Initially, the samples showed similar patterns, with differences in the intensities of some bands.

All samples exhibited a broad and intense peak at 3332 cm^−1^, which was attributed to the stretching vibrations of the –OH groups and the hydrogen bonding of cellulose [26]. The band observed in the range of 2970–2800 cm^−1^ corresponded to the stretching vibrations of C–H and CH_2_ in the cellulose molecule [27]. It can be seen that the CNF-2 samples produced two absorption bands around 2910 cm^−1^ and 2853 cm^−1^, representing symmetric and asymmetric stretching vibrations. The appearance of a single peak is due to the alignment of hydroxyl groups in the crystal lattice, resulting in the overlapping of the two peaks to form a single absorption peak. Furthermore, the absence of peaks around 1740 cm^−1^ in the LS spectra, related to the stretching vibrations of carbonyl groups (C=O) in lignin and hemicellulose [28], confirms the removal of the non-cellulosic fraction before CNF extraction. The bands at 1639 cm^−1^ are assigned to absorbed water, indicating the hydrophilic nature of cellulose [29]. Moreover, the peak at 1428 cm^−1^ is associated with CH_2_ bending vibrations and C–H stretching in cellulose. This is also known as the crystallinity band of cellulose, and thus, an increase in its intensity indicates an improvement in the degree of crystallinity [30,31]. Qualitatively, the FTIR spectra show an increase in band intensity in CNF-1 and CNF-2, which can be further confirmed by XRD studies. Asymmetric bending vibrations of C–H and C–O in the aromatic rings of the polysaccharide were recorded at 1364 cm^−1^ [32]. Moreover, the spectra of all samples exhibited typical absorption bands at 1156 cm^−1^, 1099 cm^−1^, and 1026 cm^−1^, corresponding to C–O–C glycosidic ether linkage, C–O stretching, and C–H rocking vibrations in the pyranose ring [24,33]. In terms of structure, a characteristic feature of cellulose-LS and CNF is the band present at 897 cm^−1^, associated with beta(*β*)glycosidic linkages within the monosaccharide units of cellulose. The absorption peaks in all samples indicated the same structural features of native cellulose type I, confirming that the cellulose structure was not disrupted by the treatments [34,35].

**Figure 3:**
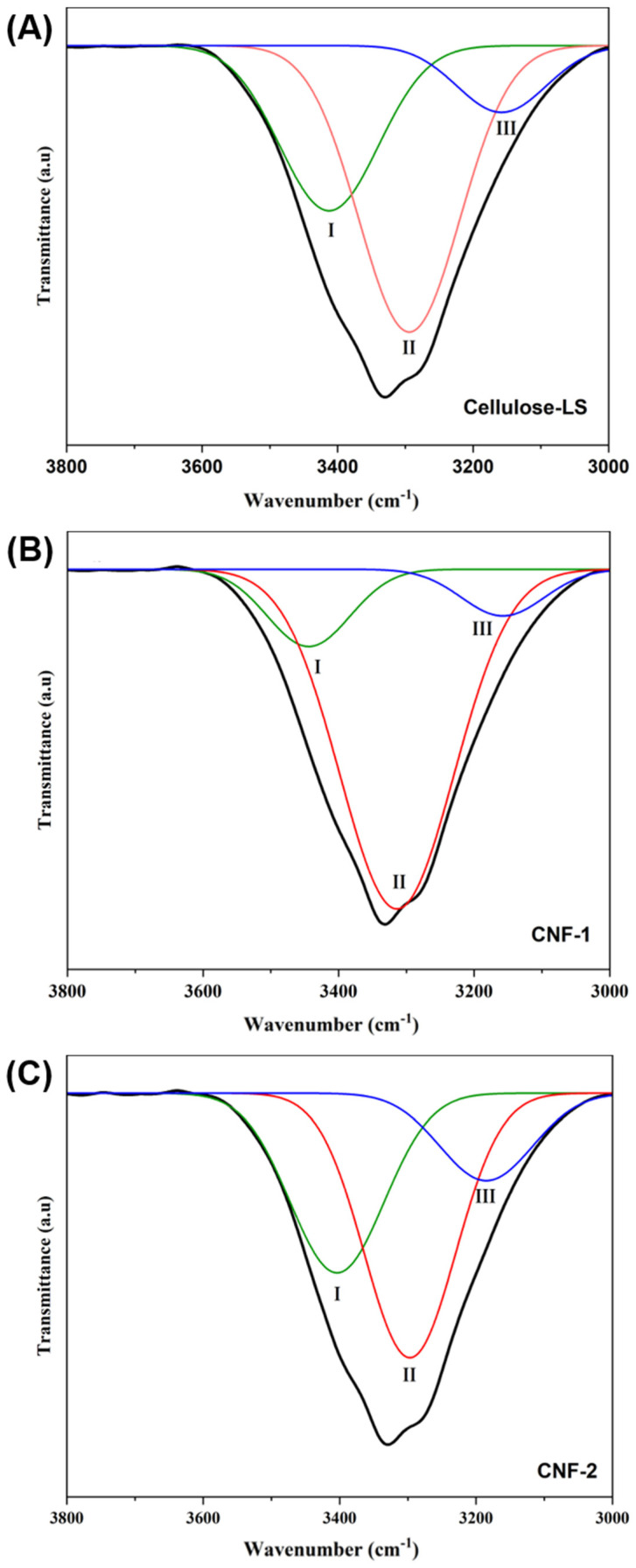
FTIR results for deconvoluted hydrogen bonding of cellulose-LS (**A**), CNF-1 (**B**), and CNF-2 (**C**) (3800–3000 cm^−1^ region). (I) O2–H⋯⋯O6, (II) O3–H⋯⋯O5, (III) O6–H⋯⋯O3.

Cellulose has a large number of hydroxyl groups, which predispose cellulose chains to form hydrogen bonds within the cellulose molecules and between the chains. As a result, two types of hydrogen bonding exist: intramolecular H-bonding provides structural rigidity, while intermolecular H-bonding holds the structure together and ensures the crystal packing of cellulose chains. To obtain information on the behavior of hydrogen bonds under different conditions and to verify the effectiveness of the treatment in fibrillating fibers to nanodimensions, the OH spectra of cellulose-LS, CNF-1, and CNF-2 were deconvoluted using the Gaussian fit in the 3800–3000 cm^−1^ region, as shown in Figure 3A–C. The deconvoluted spectra show three characteristic peaks (H-1, H-2, H-3), where H-1 and H-2 represent the intramolecular H-bond (O2–H⋯⋯O6 and O3–H⋯⋯O5), and H-3 represents the intermolecular H-bond (O6–H⋯⋯O3). The mixed modes of –OH stretching obtained correspond to the cellulose I structure [36].

Table 2 summarizes the parameters of different hydrogen bonds. The R^2^ values are greater than 0.99, indicating that the peak fitting of different hydrogen bond models can comprehensively represent the information of various such models. A closer inspection of Table 2 shows a decrease in the energy of O6–H⋯⋯O3 (intermolecular H-bonds) from cellulose-LS to CNF, while the energies of O2–H⋯⋯O6 and O3–H⋯⋯O5 (intramolecular H-bonds) increased. A possible explanation for this is the disruption of the hydrogen bonding network during the treatments. The individualization of cellulose fibers into CNF weakens the inter-chain forces of cellulose, resulting in a decrease in the bond energy. At the same time, the presence of hydroxyl groups within the nanocellulose enables the formation of intramolecular bonds, leading to an increase in bond energies. These results are consistent with a recent study by Sathwane et al. [37]. It is important to note that the intermolecular H-bond energy in CNF-2 is lower than in CNF-1. This might be explained by the swelling phenomenon.

In an alkali solution, cellulose fibers loosen their structure due to the absorption of the solvent. This swelling increases the distance between them, which weakens the intermolecular hydrogen bonds, and as a result, the energy of the intermolecular hydrogen bonds decreases. This reduction significantly contributes to the individualization of cellulose by increasing its accessibility for defibrillation to the nanoscale using mechanical or chemical methods. This facilitates the disruption of the hydrogen network with the homogenization and ultrasound treatments because the cellulose fibers are partially separated [38]. In addition, the intramolecular hydrogen bonds O3–H⋯⋯O5 are more stable than O2–H⋯⋯O6 [39].

### 3.2. X-Ray Diffraction

X-ray diffraction was used to evaluate the crystallinity structure and the alterations that occur after different chemical and mechanical treatments. This method can provide qualitative and quantitative information on the behavior of the crystalline structure. The arrangement and packing of chains in nanocellulose are key factors determining its mechanical and thermal properties, as well as its applications.

**Figure 4:**
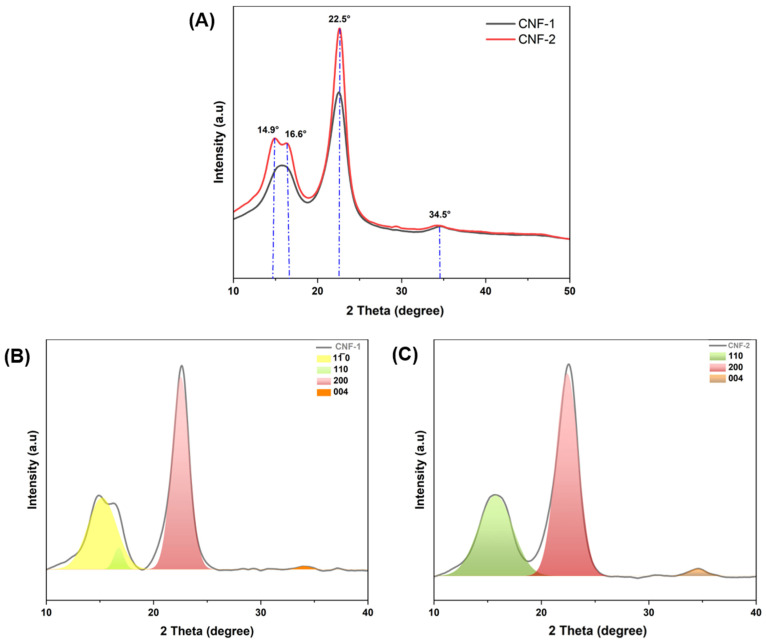
XRD patterns of (**A**) the isolated micro/nanofibers and (**B**,**C**) deconvolutions of micro/nanofibers.

The XRD patterns of CNF-1 and CNF-2 are shown in Figure 4. Notably, all of the diffractograms showed four major reflection peaks at 2θ values of approximately 14.9°, 16.6°, 22.5°, and 34.5°, corresponding to the (1$\bar{1}$ 0), (110), (200), and (004) crystallographic planes, respectively. The results indicate that the treatment preserved the typical structure of cellulose I, where the patterns combine two polymorphic forms: monoclinic and triclinic. Similar findings were observed in the nanocellulose derivatives [28,40].

The crystallinity index for cellulose fibers and the isolated CNF was calculated using the Segal high peak method [23] and peak deconvolution area [41]. Table 3 presents the crystallinity index of the samples. The *CrI* values for CNF-1 and CNF-2 improved compared with those of raw *Lygeum spartum* fibers (46.5%) [42]. This indicates the successful elimination of amorphous regions (lignin and hemicellulose) during the alkaline treatment. Generally, the mechanical method affects the crystalline structure of the cellulose nanofibers. In our case, the crystalline peak at 22.5° becomes more defined in the isolated CNF, confirming the rearrangement of the cellulose chains into an ordered structure due to the removal of amorphous cellulose [43]. These findings are in agreement with other studies, such as that of Díaz et al. [44], who reported a crystallinity index of 68% for CNF extracted from bolaina wood using microfluidization. Additionally, a study by Kaffashsaei et al. [45] reported a crystallinity index of 65% for CNF produced from the sawdust of paulownia. The most interesting observation in the XRD patterns is the appearance of two peaks at 14.9° and 16.6° in CNF-2, resulting from the change in d-spacing [46].

The calculation of crystallite size and d-spacing is shown in Table 3. The crystal size increased in CNF compared to raw fiber, as noted in our previous study [47]. The observed increase in CS may be attributed to the fact that in the cellulose crystalline regions, the chains are packed in the form of crystallites reinforced with an inter/intra-hydrogen bonding network [48]. The CS found in CNF-1 for *Lygeum spartum* (4.1 nm) was higher than that of CNF extracted from Eucalyptus wood using TEMPO-mediated oxidation (3.63 nm) [49]. Furthermore, the crystallinity and thermal stability of CNFs from *Lygeum spartum* were comparable with other CNFs extracted by other methods and prepared from the different sources [50,51].

These findings are consistent with the FTIR results. Furthermore, the interplanar spacing found in this study is close to that reported by Hernández-Becerra et al. [52], who used an anhydrous solvent for homogenization, indicating that the cellulose crystal organization structure was preserved, and certain hydrogen bonds maintained the structure. These results confirm that CS and CrI depend on the source and extraction methods.

The results of the XRD analysis revealed that the mechanical treatment has no impact on polymorphic modification. Homogenization and ultrasound generate shear forces [53] that break up the amorphous regions without significantly altering the crystalline structure. As a result, the hydrogen linkages, van der Waals forces, and molecular orientation in CNF were preserved.

### 3.3. Thermal Properties

**Figure 5:**
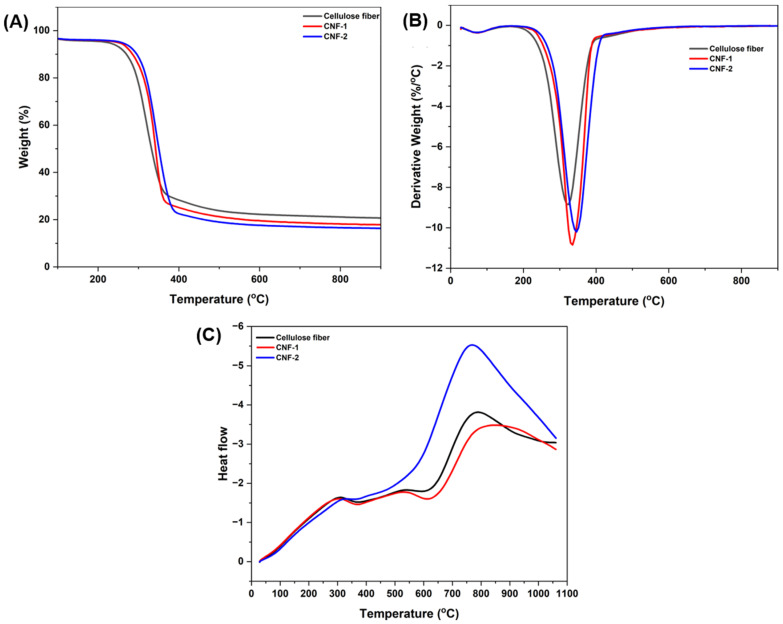
(**A**) TGA, (**B**) DTG, and (**C**) DTA curves for cellulose-LS, CNFs-1, and CNFs-2.

Thermogravimetric analysis (TGA) was used to evaluate the thermal stability and degradation properties of the isolated cellulose micro/nanofibers. The TGA, DTG (differential thermogravimetry), and DTA (differential thermal analysis) curves of cellulose fibers, CNF-1 and CNF-2, are shown in Figure 5, and the thermal data are presented in Table 4.

The thermographs indicate three stages of weight loss. In the first stage, which ranges from 28 °C to 120 °C, the weight loss is caused by the evaporation of chemisorbed water and substances of low molecular mass embedded in the fibers [54]. In the second stage, significant weight loss is observed in the 200–400 °C region. At this stage, cellulose chains and chemical linkages (C–C, C–O, and C–H) are cleaved, leading to the decomposition and degradation of the cellulose chemical structure. These results are consistent with those reported for CNF by Abraham et al. [55]. In the final stage of degradation, between 400 °C and 500 °C, a solid residue is generated. Furthermore, there are noticeable differences in thermal performance; the onset of decomposition temperatures for cellulose fiber, CNF-1, and CNF-2 are 214 °C, 225 °C, and 231 °C, respectively. The differences in crystallite size explain the variation in *T_onset_* in CNF [56]. Additionally, the *T_max_* value for CNF was validated, as shown in Table 4. This is probably due to the increase in crystallinity degree, as described in the XRD results. As shown in Table 5, CNFs have higher thermal stability compared to cellulose fibers, due to the presence of more crystalline regions, which are thermally more stable than the amorphous ones. The *T_max_* value of *Lygeum spartum* CNF-2 reported in this study is higher than that of CNF extracted from empty fruit bunches using a chemo-mechanical process (339 °C) [57], CNF isolated from pineapple leaf fibers using high-pressure homogenization and ultrasonication (320 °C) [12], and nanocellulose from cotton by acid hydrolysis (325 °C) [1]. The difference in *T_max_* for the isolated CNF is attributed to the source, the hydrogen bond network (the increase in intramolecular hydrogen bond energy), and the crystallinity index [58], which increases thermal stability.

Figure 5C shows the DTA curves of cellulose micro/nanofibers, CNF-1, and CNF-2. Differential thermal analysis reveals two endothermic peaks: the initial peak at a temperature below 100 °C corresponds to the removal of physically absorbed water, and the additional peak at 368 °C is attributed to the decomposition of the cellulose chain, with cleavage of the glycosidic linkage [59].

**Table 5:** Thermal properties of CNF fibers from Ls, and cellulosic fibers.

Sample	*T_max_* (°C)	References
CNF-1	335	Current work
CNF-2	347	Current work
*Tithonia diversifolia*	237	[60]
*Mariscus ligularis*	314	[61]
*Cissus vitiginea*	304	[62]

### 3.4. Differential Scanning Calorimetry

**Figure 6:**
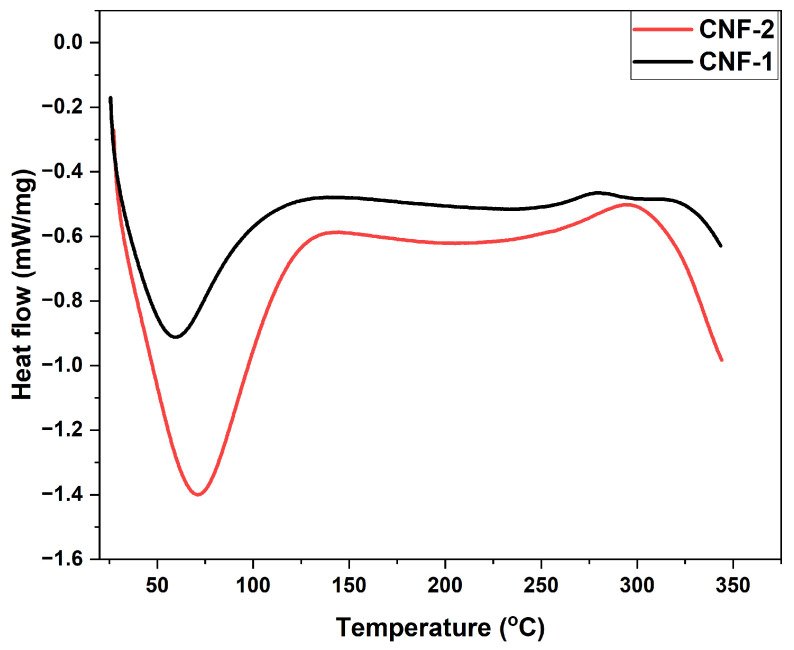
DSC thermogram of micro/nanofibers.

Differential scanning calorimetry is a thermal analysis technique that measures energy consumption and decomposition within samples. Figure 6 shows the DSC thermogram of isolated micro/nanofibers. The DSC curves exhibited both endothermic and exothermic peaks within the temperature range studied.

The first endothermic peak, observed below 100 °C, is attributed to the evaporation of surface-absorbed water; this moisture loss is validated by TGA and DTA studies. This peak is also characteristic of native cellulose [63]. A further temperature increase (˃200 °C) produces an exothermic peak in the DSC profile, which differs from the endothermic peak for cellulose nanofiber, reflecting different mechanisms during pyrolysis [64]. This indicates that pyrolysis involves both charring (exothermic) and volatilization (endothermic) processes. Another endothermic peak is observed at temperatures above 300 °C, corresponding to cellulose depolymerization and the scission of the glycosidic bonds. Yang et al. [65] reported the main mechanisms involved in cellulose decomposition: the first reaction at low temperatures generates gas (including CO, CO_2_, H_2_O), while the second reaction at higher temperatures generates organic liquids (levoglucosan).

The thermal data demonstrate that the esparto micro/nanofiber derivates have better thermal stability compared to the cellulose fibers. Thus, the homogenization and ultrasonic treatment generate forces that defibrillate and eliminate less ordered regions. As a result, the proportional size of the crystalline region increases, enhancing resistance to thermal decomposition.

### 3.5. Scanning Electron Microscopy

**Figure 7:**
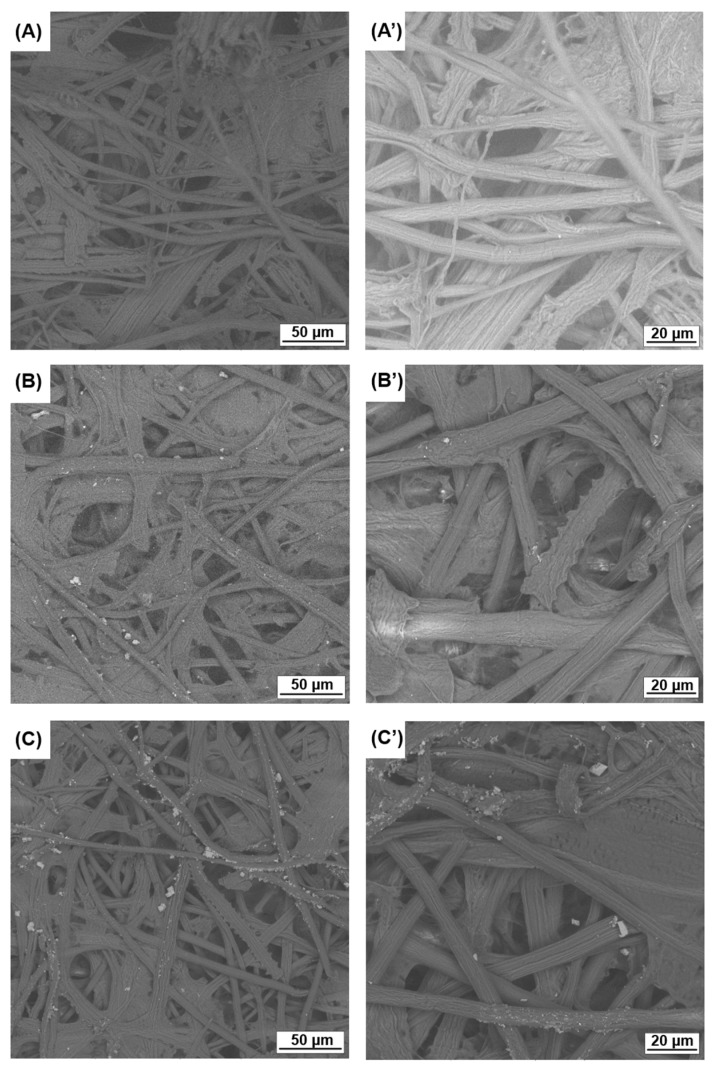
SEM images of (**A**,**A’**) cellulose-LS, (**B**,**B’**) CNFs-1, and (**C**,**C’**) CNFs-2.

Scanning electron microscopy analysis is essential to gain insight into the fiber structure and surface characteristics of *Lygeum spartum* fibers after the isolation process. Figure 7 displays the SEM micrographs of cellulose fiber, CNF-1 and CNF-2, at different magnifications.

Natural fibers are typically composed of cementing materials, including hemicellulose, lignin, waxes, and pectin, which contribute to the roughness of the fiber surface. In Figure 7A, a clear surface free from impurities is observed. The alkali treatment degraded the hemicellulose into monomers, while the lignin was depolymerized and deposited on the fiber surface. The residual lignin was eliminated during the bleaching step. These results are consistent with the functional group characterization obtained via FTIR (see Figure 2). Additionally, the fiber bundle was defibrillated into single fibers. Furthermore, the SEM images of the isolated micro/nanofibers, as shown in Figure 7B,C, revealed a network along the fiber axis. The fibrils in NC were not well individualized, due to the aggregation of the sample during the drying process. This aggregation results from the strong attraction between the hydroxyl groups, which leads to hydrogen linkages, a finding that is analogous to previous reports [66,67,68]. Chen et al. [68] used alkali–ultrasound treatment to extract CNF from windmill palms. The CNF exhibited a rod-like system, and increasing the sonication time led to the formation of a crosslinked network structure. Ultrasound induces disorder in the arrangement of fibrils, resulting in varied CNF morphologies.

**Figure 8:**
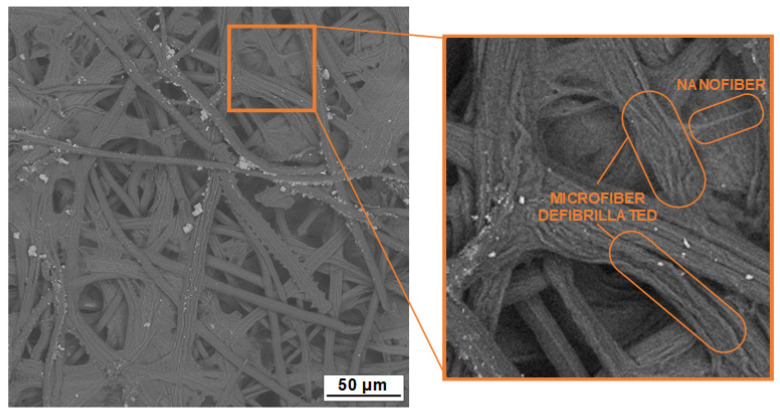
SEM image of the CNF-2 sample with indications of micro- and nanofibers.

Parts of Figure 7B,C show that the micro/nanofiber preparation method successfully defibrillated some microfiber bundles, as confirmed by the reduction in the energy of the intermolecular hydrogen bond (OH) band. Since the method is based on the use of ultrasound in the presence of water and a diluted NaOH solution, defibrillation did not reach its maximum. However, fibers can be seen detaching from the microfiber bundles, resulting in individual nanofibers (see Figure 8).

### 3.6. Atomic Force Microscopy (AFM)

**Figure 9:**
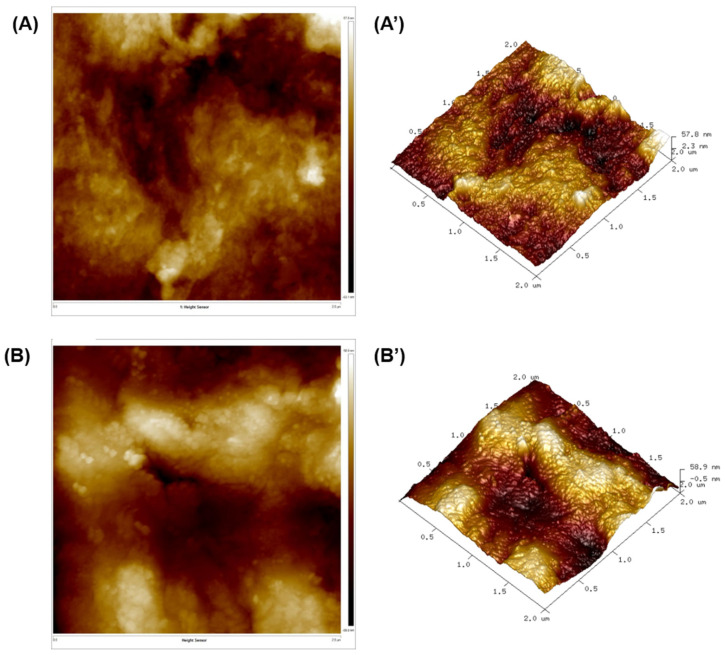
AFM images (2D, 3D) of CNFs: (**A**,**A’**) CNF-1 and (**B**,**B’**) CNF-2.

High-resolution topographic images, 3D images, and various other properties of the cellulose micro/nanofibers surfaces are shown in Figure 9. The AFM images are composed of two regions: the bright region is crystalline in nature, and the dark area is amorphous [37]. Unlike the CNC, the CNF contains an amorphous region. Figure 9 shows a long fibrillar morphology for the isolated micro/nanofiber, which is consistent with the results of the SEM analysis. Measuring the exact diameter and length of the nanofibers was challenging due to the tendency of the nanocellulose to agglomerate and the presence of hydroxyl groups on the surface, which form hydrogen bonds. This observation is consistent with a previous study by Parsai et al. [69].

Other statistical parameters, such as surface roughness (*R_a_*), kurtosis (*R_ku_*), and skewness (*R_sk_*) were measured from the height images, as shown in Table 6. The average roughness values were 2.06 and 2.86 for CNF-1 and CNF-2, respectively. The roughness is higher in CNF-2, which is favorable for adhesion to polymers in a composite. The kurtosis value (*R_ku_*) indicates the surface type: values of *R_ku_* > 3 represent a spiky surface, and values of *R_ku_* < 3 imply a rough surface. The value of *R_ku_* obtained for the isolated micro/nanofibers suggests a rough surface. The skewness value (*R_sk_*) characterizes the symmetry of the profile; a positive value indicates a porous surface, which favors the use of the materials as nanofillers in polymer matrices [25,70].

## 4. Conclusions

Cellulose micro/nanofibers were successfully isolated from *Lygeum spartum* using a green multi-step approach combining alkali treatment, ultrasonication, and homogenization. The chemical, microstructural, thermal, and morphological properties of the processed micro/nanofibers were investigated to gain better insight into the process efficiency. The conversion of *Lygeum spartum* to micro/nanofibers began with the removal of hemicellulose, lignin and other extractives by chemical treatment, which was confirmed by FTIR analysis. Additionally, the X-ray diffraction analysis revealed the presence of cellulose I and an increase in the crystallinity index, attributed to the removal of amorphous regions, mainly the amorphous cellulose, where the hydrogen linkages have lower energy and can be disrupted by the mechanical forces during homogenization and sonication treatments. Thermal stability was comprehensively investigated using thermogravimetric analysis. The TGA, DTA, and DSC curves showed that the micro/nanofibers from *Lygeum spartum* had impressive thermal stability. It is important to note that using a dilute alkali solution produces micro/nanofibers with a higher crystallinity index and enhanced thermal stability compared to the product obtained using water as a solvent. The SEM images showed fibers free of impurities and waxy substances, and the formation of fibers along the axis, suggesting better mechanical properties of the nanocomposites. The AFM analysis confirmed a fibrillar morphology of the cellulose micro/nanofibers, with a rough surface. The experimental results of this study validate the possibility of extracting cellulose micro/nanofibers from new resources, and the knowledge gained from the different analyses supports the valorization of *Lygeum spartum* grass. The ultrasound method is characterized by its frequency parameter. The use of this method for the defibrillation of microstructures (microfibers) in the presence of strong hydrogen bond interactions has demonstrated its effectiveness in weakening the interactions between fibers, as confirmed by the FTIR results. From a morphological perspective, this method also defibrillated some fibers at the nanometric scale.

Future studies will focus on the investigation of the mechanical properties of micro/nanofibers and their use as an additive in recycled paper, as well as their application as reinforcement in composites.

## Figures and Tables

**Table 2:** Parameters of H-bonding.

Sample	H-Bond	Wavenumber (cm^−1^)	Binding Energy, *E_H_* (kcal)	Bond Length, *R* (nm)	*R* ^2^
Cellulose-LS	O2—H⋯⋯O6 ^a^	3427	2.86	0.28	0.99
	O3—H⋯⋯O5 ^a^	3330	4.46	0.27	
	O6—H⋯⋯O3 ^b^	3172	7.07	0.27	
CNF-1	O2—H⋯⋯O6 ^a^	3452	2.44	0.28	0.99
	O3—H⋯⋯O5 ^a^	3317	4.67	0.27	
	O6—H⋯⋯O3 ^b^	3216	6.34	0.27	
CNF-2	O2—H⋯⋯O6 ^a^	3415	3.05	0.28	0.99
	O3—H⋯⋯O5 ^a^	3331	4.44	0.27	
	O6—H⋯⋯O3 ^b^	3205	6.53	0.27	

^a^ Intramolecular hydrogen bonding. ^b^ Intermolecular hydrogen bonding.

**Table 3:** Crystal parameters (crystallinity, crystal size (CS), and d-spacing) of CNF-1 and CNF-2.

Sample	Crystallinity (%)		CS (nm)	D-Spacing (nm)
	Segal Method	Peak Deconvolution		
CNF-1	69.5	59.6	4.1	3.9
CNF-2	75.0	61.1	3.3	3.4

**Table 4:** Thermal properties data for cellulose-LS, CNF-1, and CNF-2.

Sample	*T_onst_* (°C)	*T_max_* (°C)	Residue (%)
Cellulose-LS	214	323	20.3
CNF-1	225	335	17.6
CNF-2	231	347	16.1

**Table 6:** AFM analysis parameters.

Sample	*R_a_* (nm)	*R_sk_*	*R_ku_*
CNF-1	17.4	0.054	2.06
CNF-2	13.2	0.205	2.86

## Data Availability

The original contributions presented in the study are included in the article, further inquiries can be directed to the corresponding authors.
